# High sensitivity carbon monoxide detector using iron tetraphenyl porphyrin functionalized reduced graphene oxide

**DOI:** 10.1186/s11671-023-03813-9

**Published:** 2023-03-07

**Authors:** Sumedh M. Shirsat, Chih-Hao Chiang, Gajanan A. Bodkhe, Mahendra D. Shirsat, Meng-Lin Tsai

**Affiliations:** 1 Department of Materials Science and Engineering, National Taiwan University of Science and Technology, Taipei City, 106335 Taiwan; 2 Department of Food Science and Technology, Yeungnam University, Gyeongsan, Gyeongsangbuk-do 38541 Republic of Korea; 3 Department of Physics, RUSA Center for Advanced Sensor Technology, Dr. Babasaheb Ambedkar Marathwada University, Aurangabad, MS 431004 India

**Keywords:** Reduced graphene oxides, Tetraphenyl porphyrins, Iron tetraphenyl porphyrins, Carbon monoxide, Gas sensors

## Abstract

The detection of pollutant and toxic gases has attracted extensive attention due to the growing environmental issues. In the present investigation, free-based tetraphenyl porphyrin (TPP) and iron tetraphenyl porphyrin (FeTPP) are used to functionalize thermally reduced graphene oxide (rGO) and further used for the detection of carbon monoxide (CO). TPP and FeTPP functionalized rGO (FeTPP@rGO) sensors are fabricated on a glass substrate with thermally coated copper electrodes. The materials are characterized with X-ray diffraction (XRD), Fourier transforms infrared (FTIR) spectroscopy, Raman spectroscopy, UV–visible spectroscopy, atomic force microscopy, scanning electron microscopy, and energy dispersive spectroscopy. The current–voltage (*I*–*V*) characteristics have also been studied to demonstrate the operation of the device. In addition, the FeTPP@rGO device shows high sensitivity toward the detection of CO. By testing in the chemiresistive sensing modality, the as-fabricated device shows good response and recovery of 60 s and 120 s, respectively, with a low detection limit of 2.5 ppm.

**Supplementary Information:**

The online version contains supplementary material available at 10.1186/s11671-023-03813-9.

## Introduction

An incomplete combustion of carbon results in the formation of carbon monoxide (CO), which is usually found in exhaust emissions from vehicles and household environments. It is a colorless, odorless, undesirable, noxious, and toxic gas that permeates our environment. The continuous exposure to CO can result in headache, vomiting, loss of consciousness, muscle weakness, or nausea, which are symptoms of CO poisoning. To prevent these unfortunate accidents, designing low-cost and portable gas sensors to detect CO in daily lives is highly demanded.

Various researchers across the globe have been investigating numerous materials to achieve optimal sensing performance from gas sensors for the detection of CO [1–6]. For example, in recent years, carbon-based materials such as graphene and carbon nanotubes (CNTs) have stepped forward as superior materials in the gas sensing aspect due to their high surface-to-volume ratio and exceptional electrical properties [7–14]. Reduced graphene oxide has often been used for sensing applications because of its semiconducting nature instead of pristine graphene, which exhibits conducting characteristics [15–19]. Over the years, graphene deposition techniques have evolved to show the industrial applicability of graphene. Graphene sheets prepared using mechanical exfoliation, chemical vapor deposition (CVD), and reduced graphene oxide (rGO) sheets by Hummers method have been widely used for gas sensing applications [20–27]. In the case of graphene chemiresistors, intrinsic graphene sheets have almost no selectivity [25–28]. Therefore, graphene functionalization has been proposed to overcome this barrier. Different interaction strengths between analytes and functional species would determine the selective detection as they could preferentially anchor a required target gas. Various functional materials such as conducting polymers, organometallic molecules, metal oxides, and metal nanoparticles have been used to enhance sensing performance by improving sensitivity, recovery time, and specifically selectivity, which has always been a significant drawback for carbon-based gas sensors [29–36]. Porphyrins are aromatic macrocyclic rings consisting of four pyrrole-type rings in a conjugated system. In addition, they interact with target analyte through π-π stacking, which does not affect carbon hybridization and intrinsic electronic transport properties [28]. Therefore, the chemical response can be further improved by replacing the two central hydrogen atoms with a transition metal atom to obtain the metalloporphyrin, which exhibits significant catalytic properties.

In the present study, we demonstrate a drastic change in sensitivity toward CO in a metalloporphyrin functionalized rGO sensor. Graphene oxide was thermally reduced at an optimized temperature and further functionalized via tetraphenyl-porphyrin and tetraphenyl-porphyrin iron(III) chloride. By adopting the metalloporphyrin functionalized rGO sensor, an explosive sensitivity toward CO has been successfully achieved. These fascinating results show that the fabricated gas sensor would be feasible for practical CO detection applications.

## Methods

### Graphene oxide (GO) synthesis

GO was synthesized via the oxidation of graphite using the Hummers method [37]. Concisely, graphite powder (1.2 g) and NaNO_3_ (2 g) were mixed with H_2_SO_4_ (50 ml). This mixture was kept in an ice bath with continuous stirring for 2 h as KMnO_4_ (6 g) was added to the suspension very slowly by carefully controlling the rate of addition to maintain the temperature below 14 °C. Further, the ice bath was removed, and the mixture was stirred for another 2 h at 35 °C until it became partly brownish, assisted with the slow addition of deionized (DI) water (100 ml), causing the reaction temperature to be swiftly increased (98 °C) with effervescence, which in turn changed the mixture to brownish color. Under constant stirring, additional DI water (200 ml) was added under continuous stirring to further dilute the solution. It is then treated with H_2_O_2_ (8 ml) to terminate the reaction, turning the solution partly yellowish. GO was purified via centrifugation and rinsed with HCl (8%) and DI water various times until the pH value was around 7. It was further processed via filtering and drying in a hot air oven to obtain GO in powder form and reserved for further use.

### GO, TPP, and FeTPP solution preparation

10 mg/ml of GO was prepared in deionized water and ultrasonicated for 90 min. A 0.1 mM TPP suspension was prepared in 10 ml of DMF by dispersing 0.614 mg of 5,10,15,20-Tetraphenyl-21H,23H-porphine and further ultrasonicated for 10 min. Similarly, a 0.01 mM FeTPP suspension was prepared in 10 ml of DMF by dispersing 0.704 mg of 5,10,15,20-tetraphenyl-21H,23H-porphine iron (III) chloride and further ultrasonicated for 10 min.

### Characterizations

The transmittance spectra of studied materials were characterized using Fourier transform infrared (FTIR) spectroscopy on a Bruker Alpha FTIR with an attenuated total reflection (ATR) attachment in the 600–4000 cm^−1^ range. The absorption spectra of the samples were measured using a Jasco V-750 spectrometer in the 190–900 nm range. Raman spectroscopy was studied on a XploRA PLUS Raman spectrometer (Horiba, France) with a 50 × objective. X-ray diffraction (XRD) patterns were investigated using a Bruker D8 Advance Diffractometer with a CuKα radiation source (*λ* = 1.5418 Å). Morphological characterization was carried out using a Park XE-7 atomic force microscope (AFM) and a TESCAN MIRA3 field emission scanning electron microscope (SEM). AFM image analysis was carried out in XEI software (1.8.0 version) provided by PARK Systems, Korea. Elemental analysis was carried out by energy dispersive spectroscopy (EDS). Electrical characterizations (*I*–*V*) and chemiresistive sensing experiments were measured with a Keithley 4200A semiconductor analyzer at room temperature (22 °C) in an indigenously designed and fabricated dynamic gas sensing system.

### Sensor fabrication and thermal reduction of graphene oxide

**Fig. 1:**
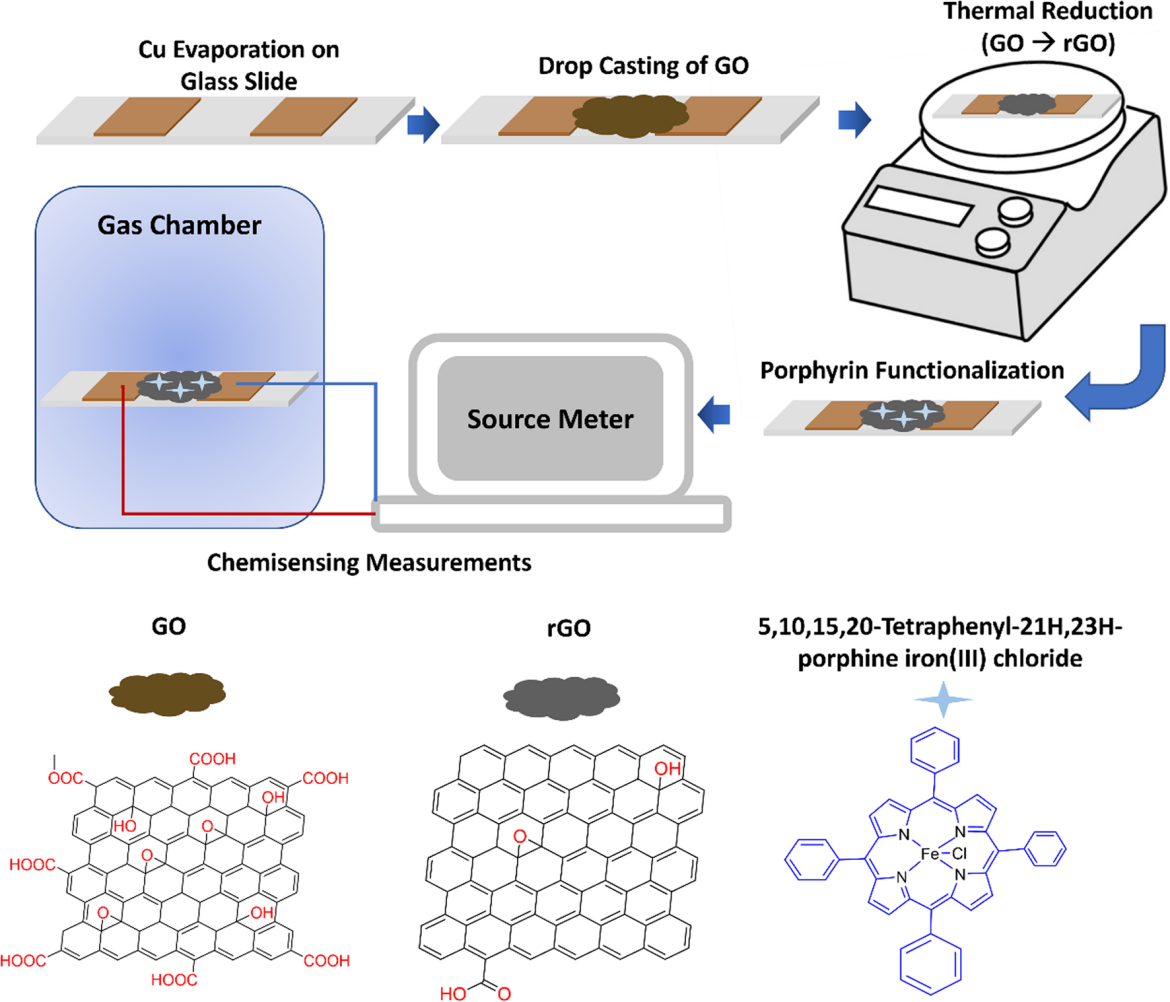
Schematic representation of experimental procedures along with the molecular structures of rGO and FeTPP

As shown in Fig. 1, glass slides were cleaned with acetone and isopropyl alcohol and then coated with copper via thermal evaporation with shadow mask to obtain patterned copper electrodes. GO was then drop casted between the two electrodes, after which these chips were heated at 200 °C on a hotplate for 5 min, resulting in the thermal reduction of GO to obtain rGO. TPP and FeTPP were then drop casted onto two similar rGO chips and dried at room temperature (22 °C).

### Gas sensing measurements

The changes in resistance caused by the influence of CO gas and experimental conditions were measured using a home-made dynamic gas sensing setup. The gas chamber was crafted using a glass of 8-cc volume with inlet and outlet gas ports. Additionally, the calibrated concentration of testing gas kept in Tedlar bags was utilized to inject CO gas, maintaining a flow rate of 200 sccm. A program-controlled script was run using Alicat Mass Flow Controllers (MFCs) to achieve the target concentrations, while dry air was used as reference gas for sensing measurement. The sensors were sustained under the steady flow of dry air for 5 min before the target gas with various concentrations was exposed to the sensor chip for the script duration. Chemisensing measurements were characterized using a Keithley 4200A semiconductor parameter analyzer.

## Results and discussion

### FTIR, Raman, UV–visible, and XRD characterizations

**Fig. 2:**
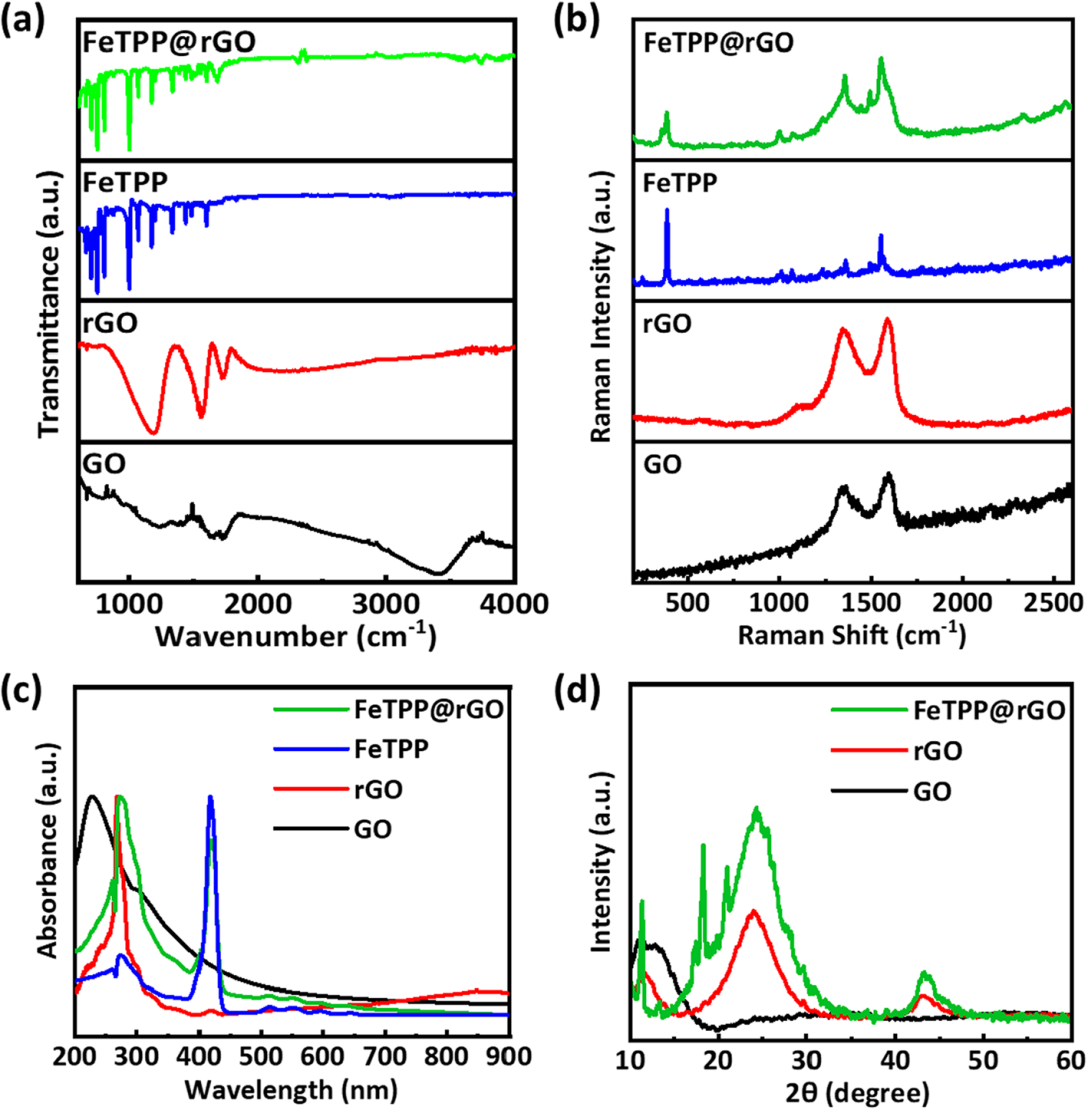
**a** FTIR spectra of GO, rGO, FeTPP, and FeTPP@rGO. **b** Raman spectra of GO, rGO, FeTPP, and FeTPP@rGO. **c** UV–visible spectra of GO, rGO, FeTPP, and FeTPP@rGO. **d** XRD patterns of GO, rGO, and FeTPP@rGO

Figure 2a shows the FTIR spectra of GO, rGO, FeTPP, and rGO@FeTPP. The GO spectrum exhibits the presence of bands associated with C–O–H, C=C, and C=O stretching modes at 1043 cm^−1^, 1632 cm^−1^, and 1716 cm^−1^, respectively [23]. The broad peak of GO between 3600 and 3000 cm^−1^ corresponds to the hydroxyl group (O–H). The GO spectrum shows peaks at 1627 cm^−1^ and 1020 cm^−1^, which confirms the presence of carbonyl (C=O) and carboxyl (COOH) groups, respectively. The absence of the O–H broad peak of the carboxyl group confirms the GO is partially reduced after the thermal treatment. The FTIR spectra of FeTPP and rGO@FeTPP show the presence of transmittance peak at 1022 cm^−1^, indicating the presence of Fe-porphyrin in both FeTPP and rGO@FeTPP. The bands between 650–820 cm^−1^ and 960–1220 cm^−1^ have been reported to correspond to benzene skeleton stretching, C–H out-of plane bending and in-plane bending vibrations of benzene, respectively [38]. Characteristic peaks containing functional groups such as C–O–H, C=C, C=O were retained in FeTPP@rGO spectrum, indicating the effective functionalization of rGO. Figure 2b shows the Raman spectra of GO, rGO, FeTPP, and rGO@FeTPP. The structural changes of GO and rGO were observed through Raman spectroscopy. The two characteristic bands are observed at 1347 cm^−1^ (D band) and 1587 cm^−1^ (G band), respectively. The D band reveals the degree of disorder in GO and rGO. The intensity of the D band is increased after thermal reduction of GO, and the appearance of the G band is due to the optical E_2g_ phonons at the Brillouin zone center, which results in stretching of sp^2^ carbon networks [39]. The strong band at 390 cm^−1^ and the weak band at 200 cm^−1^ are the two lowest frequency A_g1_ modes of the TPP [40]. A significant portion of the Raman spectrum of FeTPP matches with the rGO@FeTPP. The distortion in the composite spectra near 1350 cm^−1^ and 1590 cm^−1^ indicates the presence of the characteristic D and G bands of rGO, respectively. Figure 2c shows the UV–visible spectra of GO, rGO, FeTPP, and rGO@FeTPP. The absorption peak at 232 nm in the GO spectrum is due to the π-π* transitions of the aromatic C–C bonds and the peak at 300 nm due to n–π* transitions of C=O bonds. After the thermally reducing GO to form rGO, the absorption peak is red shifted to ~ 270 nm due to the completion of the deoxygenation process. The peak at 420 nm is the characteristic peak of FeTPP. The presence of similar absorption peaks from rGO and FeTPP in rGO@FeTPP spectrum imply that functionalization has been realized [41]. Figure 2d shows the XRD patterns of GO, rGO, and rGO@FeTPP, the intense peak at the 2*θ* value 11.09° is due to the (001) plane whereas the broad peak at 2*θ* value 25° indicates the exploitation of the graphene sheet during thermal treatment [22]. The XRD pattern of FeTPP@rGO shows the FeTPP is polycrystalline and monophasic in nature and the hump near 20° indicates the presence of rGO [42].

### Atomic force microscopy (AFM) and scanning electron microscopy (SEM) analysis

**Fig. 3:**
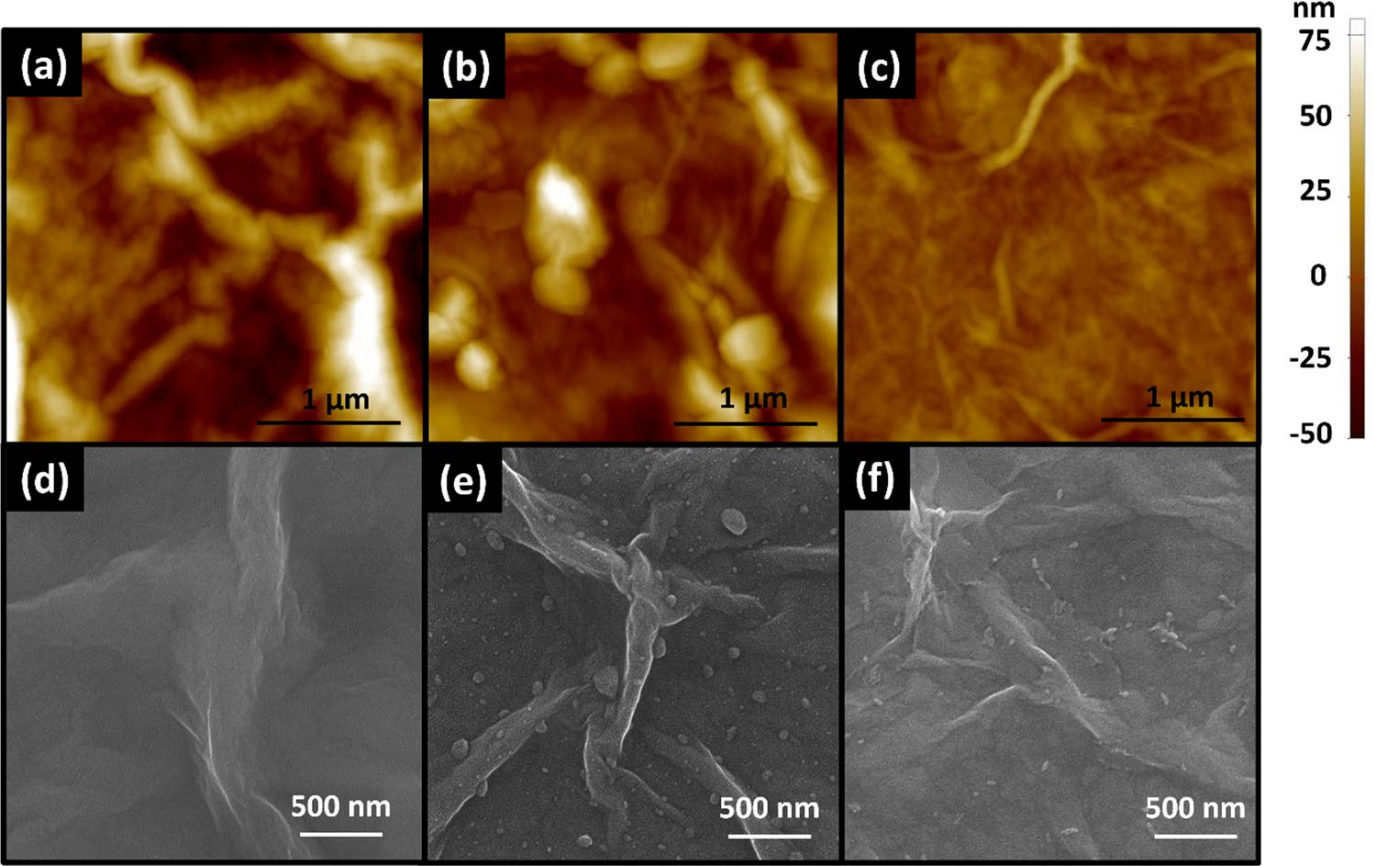
**a** AFM image of the GO film. **b** AFM image of the rGO film. **c** AFM image of the FeTPP@rGO film. **d** SEM image of the GO film. **e** SEM image of the rGO film. **f** SEM image of the FeTPP@rGO film

The surface morphologies of GO, rGO, and FeTPP@rGO have been investigated by atomic force microscope (AFM) and are shown in Fig. 3a–c, respectively. The morphological information of the samples is listed in Table 1. It is observed that after the thermal reduction process, the surface area, root mean square roughness (Rq), and average roughness are decreased (Table 1), which confirms the reduction of GO. The increase in surface area ratio (%) can be calculated with

1 $${\text{Increase}}\;{\text{in}}\;{\text{surface}}\;{\text{area }}\;{\text{ratio}} \left( \% \right) = \frac{{A_{{\text{S}}} - A_{{\text{G}}} }}{{A_{{\text{G}}} }} \times 100\%$$

where *A*_S_ and *A*_G_ represent surface area and geometric area, respectively. Moreover, after functionalization of rGO by FeTPP, the AFM image of the FeTPP@rGO film clearly shows a relatively featureless surface with further decrease in roughness which confirms the functionalization of rGO by FeTPP. The surface morphology of GO, rGO, and FeTPP@rGO has also been investigated by SEM and is shown in Fig. 3d–f, respectively. In addition, the EDS spectrum (Additional file 1: Fig. S1) of FeTPP@rGO has also revealed the presence of iron (Fe), indicating the presence of FeTPP on the surface of the rGO.

**Table 1:** AFM morphological information

Material	Geometric area (µm^2^)	Surface area (µm^2^)	Increase in surface area ratio (%)	Root mean square roughness (nm)	Average roughness (nm)
GO	9	9.35	3.9	30.0	23.9
rGO	9	9.25	2.8	20.9	16.5
FeTPP@rGO	9	9.18	2.0	8.5	6.4

### Sensor performance

**Fig. 4:**
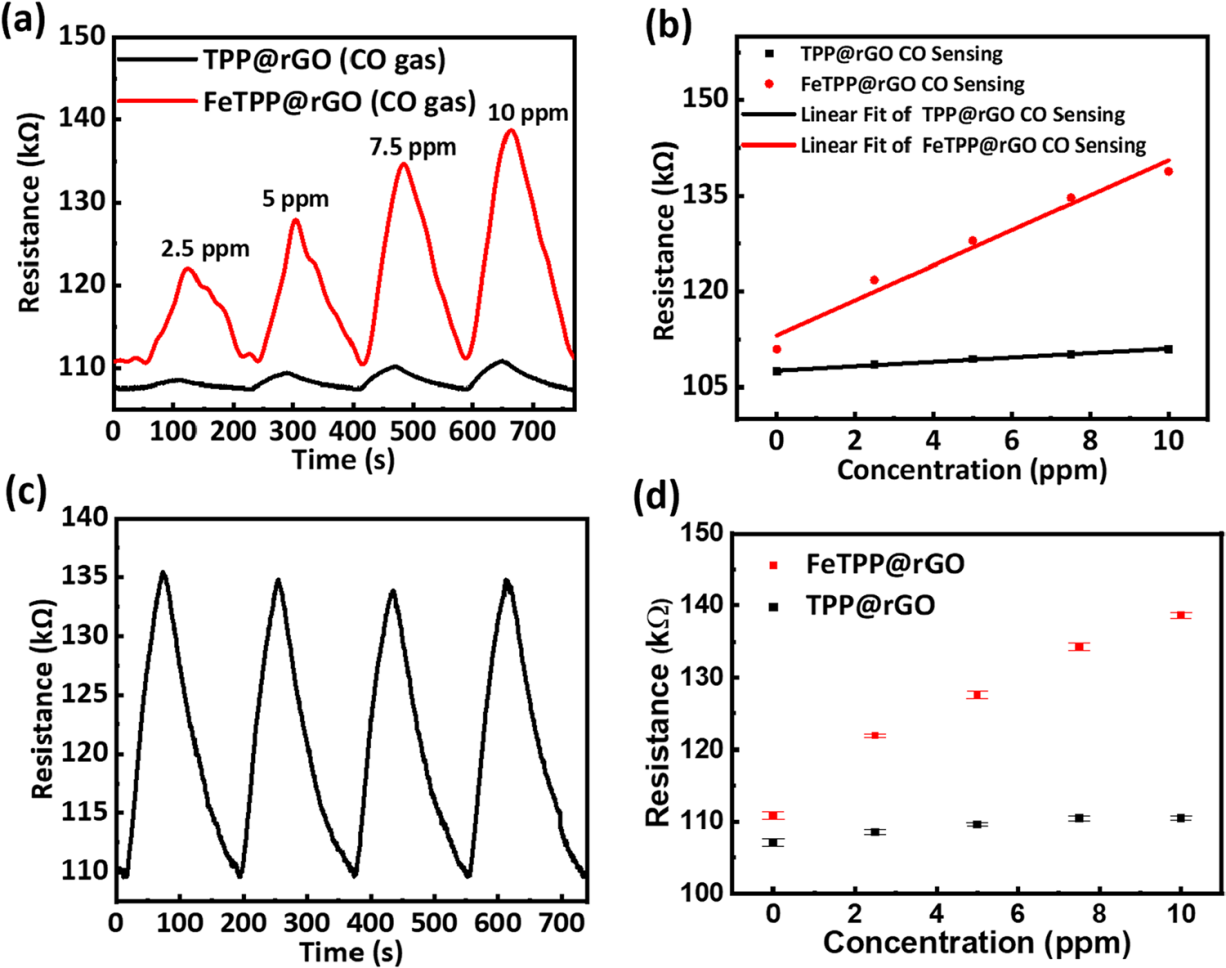
**a** Comparative sensing response of TTP@rGO and FeTPP@rGO devices for CO gas. **b** Calibration plot of TPP@rGO and FeTPP@rGO devices for CO gas. **c** Repeatability of FeTPP@rGO sensing device at 7.5 ppm of CO gas. **d** Error bar plot of 5 measurements of TPP@rGO and FeTPP@rGO sensing devices for various concentrations of CO gas

The electrical behavior of rGO and FeTPP@rGO films was investigated at room temperature (22 °C) and is shown in Additional file 1: Fig. S2. An increase in electrical resistance of rGO samples after functionalization with FeTPP can be observed. This phenomenon occurs due to forming a charge-transfer complex between rGO and FeTPP as the hexatomic ring of carbon atoms and the aromatic planar structure of porphyrin can be spontaneously stacked with each other via the π–π interactions, giving rise to a higher resistance than that of rGO. The electron-donating nature of FeTPP can be expected, resulting in a decrease in the charge carrier (hole) concentration of rGO. To evaluate the CO response of FeTPP@rGO, chemisensing devices based on TPP@rGO and FeTPP@rGO have been fabricated and characterized in the gas chamber. The chemiresistive sensing performances of TPP@rGO and FeTPP@rGO devices are shown Fig. 4a, b. A response at 2.5 ppm concentration was observed for CO on both TPP@rGO and FeTPP@rGO devices, well below the Occupational Safety and Health Administration (OSHA) permissible exposure limits (PELs) of 50 ppm. Thus, we conclude that these sensors can be used to detect CO in extreme conditions. However, it can be clearly observed that FeTPP@rGO exhibits significantly higher sensing response toward CO as compared to TPP@rGO. Here the sensing response is evaluated as the change in resistance (R) in the presence of electron-donating CO gas. Moreover, it can be clearly seen from Fig. 4b that the calibration plot of FeTPP@rGO sensor is more linear as compared to TPP@rGO sensor. This indicated that the sensitivity of FeTPP@rGO sensor is higher compared to TPP@rGO sensor.

**Fig. 5:**
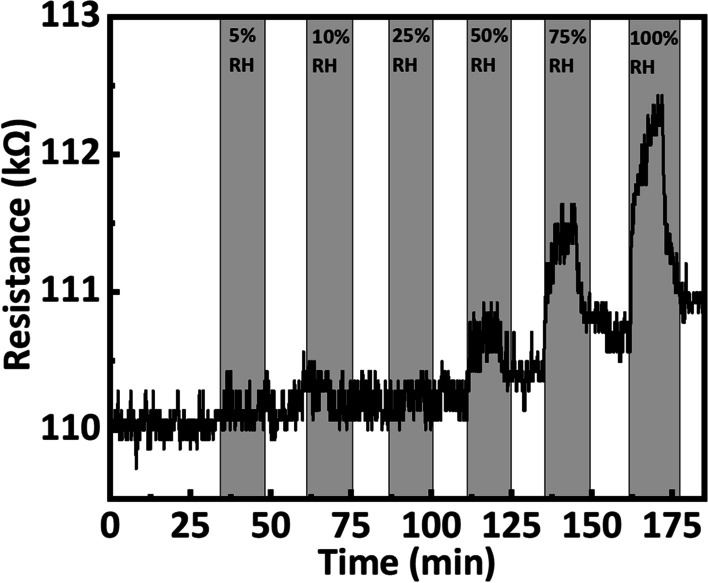
Relative humidity response for FeTPP@rGO sensor

A charge-transfer complex due to π–π stacking can be formed as FeTPP interacts with rGO after functionalization. Since metalloporphyrins are well-known electron donors and rGO is a p-type material, FeTPP will modulate the conductivity after functionalization and enhance the investigated chemical response due to its strong catalytic activity. The fabricated sensors show good repeatability and reproducibility, as shown in Fig. 4c, d, respectively. It is observed that the sensor shows response and recovery time of 60 s and 120 s, respectively. The repeatability of the fabricated sensor device toward CO gas shows less than 1% decrease in response at 7.5 ppm. Also, the sensor does not show any response up to 25% relative humidity and change in resistance is very small above 50% of relative humidity as compared to the CO gas response as shown in Fig. 5. As shown in Table 2, we have summarized a comprehensive comparison of sensing properties including sensing modality, limit of detection (LOD), response time, and recovery time of our work with previously studies. It shows that FeTPP@rGO exhibits improved performance in terms of LOD and response time under room temperature (22 °C) condition compared to previous works [43].

**Table 2:** A comprehensive comparison of CO sensing properties of graphene-based gas sensors

No	Material	Temperature (°C)	LOD	*T*_res_	*T*_rec_	References
1	FeTPP@rGO	RT	2.5 ppm	60 s	120 s	This work
2	Graphene	20	100 ppm	70 s	40 s	[44]
3	ZnO–Graphene	RT	22 ppm	10 min	10 min	[45]
4	Graphene (Films & Ribbons)	RT	100 ppm	~ 15 min	~ 15 min	[46]
5	WO_3_:graphene (2:1)	300	10 ppm	16 min	~ 4 min	[47]
6	ZnO–Graphene	RT	10 ppm	280 s	45 s	[48]
7	Grapene-CeO_2_ (Nanoplatelets)	RT	2 ppm	100 s	50 s	[49]
8	Co(III) doped-CoFe LDH/GO	RT	0.002 mg/L	0.5 s	1 s	[50]

## Conclusions

In conclusion, TPP@rGO and FeTPP@rGO chemiresistive devices have been fabricated, characterized, and tested for the detection of CO gas analyte. After FeTPP functionalization, the uniformity of the film has been enhanced. Due to the high catalytic activity of the metalloporphyrin, π–π stacking induced after the functionalization, and charge-transfer complex formed as FeTPP interacts with rGO, the FeTPP@rGO shows significantly improved sensitivity with good repeatability, reproducibility, and excellent LOD toward CO gas. The scheme combining metalloporphyrin and rGO demonstrated herein provides the potential for the design of high-performance gas sensing related applications.

## Supplementary Information

**Additional file 1: Fig. S1.** EDS spectrum of FeTPP@rGO. **Fig. S2.** Current-voltage characteristics of rGO and FeTPP@rGO.

## Data Availability

All data generated or analyzed during this study are included in this published article and its supplementary information files.

## Abbreviations

TPP: Tetraphenyl porphyrin
FeTPP: Iron tetraphenyl porphyrin
rGO: Reduced graphene oxide
CO: Carbon monoxide
XRD: X-ray diffraction
FTIR: Fourier transforms infrared
AFM: Atomic force microscopy
SEM: Scanning electron microscopy
EDS: Energy dispersive spectroscopy
CNTs: Carbon nanotubes
CVD: Chemical vapor deposition
ATR: Attenuated total reflection
MFCs: Mass flow controllers
OSHA: Occupational Safety and Health Administration
PELs: Permissible exposure limits
LOD: Limit of detection
