# Supplementary material for: High sensitivity carbon monoxide detector using iron tetraphenyl porphyrin functionalized reduced graphene oxide
Source: Discov Nano. 2023 Mar 7;18(1):34. doi: 10.1186/s11671-023-03813-9 (PMC9992485; doi:10.1186/s11671-023-03813-9)
Supplement: Supplementary file 1 — Additional file 1: Fig. S1. EDS spectrum of FeTPP@rGO. Fig. S2. Current-voltage characteristics of rGO and FeTPP@rGO. [file 11671_2023_3813_MOESM1_ESM.docx]

**Supplementary Material**

**High Sensitivity Carbon Monoxide Detector Using Iron Tetraphenyl Porphyrin Functionalized Reduced Graphene Oxide**

Sumedh M. Shirsat ^1^, Chih-Hao Chiang ^1^, Gajanan A. Bodkhe ^2^, Mahendra D. Shirsat ^3^ and Meng-Lin Tsai ^1, *^

*^1^ Department of Materials Science and Engineering, National Taiwan University of Science and Technology, Taipei City 106335, Taiwan*

*^2^* *Department of Food Science and Technology, Yeungnam University, Gyeongsan, Gyeongsangbuk-do 38541, Republic of Korea*

*^3^ RUSA Center for Advanced Sensor Technology, Department of Physics, Dr. Babasaheb Ambedkar Marathwada University, Aurangabad, MS 431004, India*

*E-mails: [mltsai@mail.ntust.edu.tw](mailto:mltsai@mail.ntust.edu.tw)


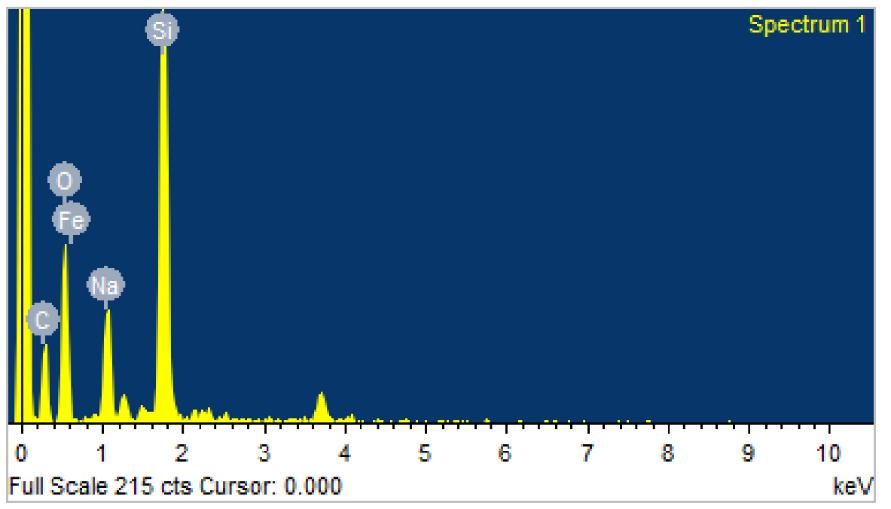


**Figure S1. EDS spectrum of FeTPP@rGO.**


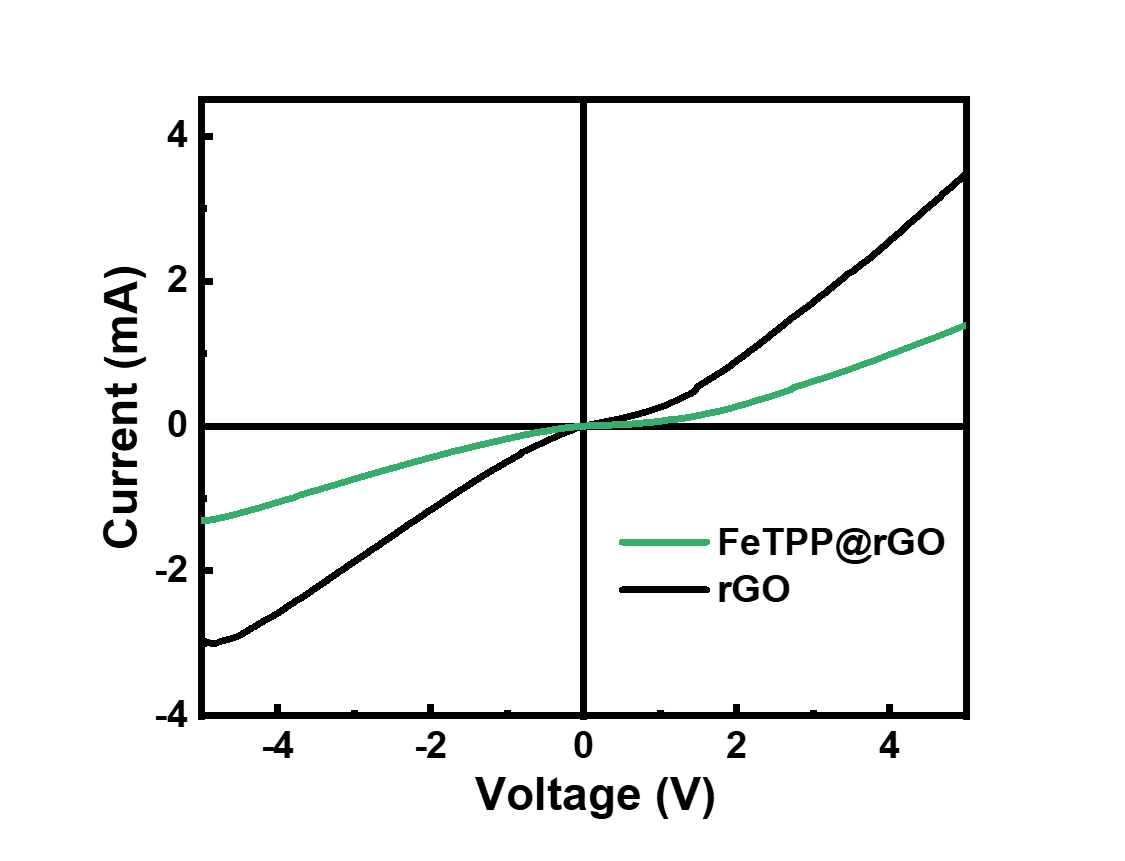


**Figure S2. Current-voltage characteristics of rGO and FeTPP@rGO.**
